# The Role of Eyelid Margin Vascularity in Meibomian Gland Dysfunction Diagnosis and Management

**DOI:** 10.7759/cureus.93587

**Published:** 2025-09-30

**Authors:** Nikolaos Kappos, Ilias Georgalas, Dimitrios Papaconstantinou, Konstantinos Droutsas

**Affiliations:** 1 Department of Ophthalmology, Naval Hospital of Athens, Athens, GRC; 2 First Department of Ophthalmology, National and Kapodistrian University of Athens, Athens, GRC

**Keywords:** dry eye, eyelid margin, meibomian gland dysfunction, telangiectasia, vascularity

## Abstract

This review investigates the diagnostic and prognostic significance of telangiectatic meibomian gland dysfunction (MGD), as well as potential therapeutic approaches. A thorough scientific literature search was conducted using various databases. Studies focusing on eyelid vascular changes in MGD published between 2010 and 2024 were reviewed. Paper selection was based on their contribution to the understanding of the pathophysiology, diagnosis, and management of the disease. Ocular surface inflammation, oxidative stress, and cytokine activity are the main molecular pathways involved in the pathophysiology of MGD, which leads to increased eyelid margin vascularity. Newer imaging techniques, especially swept-source optical coherence tomography angiography (SS-OCTA), have enabled quantification and objective visualization of this pathology. Additionally, the classification of MGD into telangiectatic and non-telangiectatic forms aids in treatment customization. Promising treatments targeting vascular abnormalities, such as intense pulsed light (IPL), anti-vascular endothelial growth factor (VEGF) agents, and cyclosporine, have emerged to improve meibomian gland function and reduce inflammation. Eyelid margin vascularity is an important biomarker for the diagnosis and management of MGD. With improved imaging techniques and targeted treatments, clinicians can more precisely evaluate disease severity and offer more effective interventions. Further research is needed to standardize diagnostic criteria, customize treatment protocols, and investigate new therapeutic targets for clinical management.

## Introduction and background

Meibomian gland dysfunction (MGD) is considered the leading cause of evaporative dry eye disease (DED), as its prevalence varies from 3.5% to 70% [1]. DED, characterized by rapid tear evaporation due to a deficient lipid layer, causes chronic discomfort, including burning, itching, and blurred vision, significantly impacting patients' quality of life. The International Workshop on MGD defines the disease as a chronic diffuse abnormality of the meibomian glands, commonly characterized by terminal duct obstruction and/or qualitative/quantitative changes in gland secretion. It can result in alteration of the tear film, symptoms of eye irritation, clinically apparent inflammation, and ocular surface disease [2]. Clinical signs of MGD affecting eyelid margin are eyelid thickening, notching of eyelid margin, uneven tear meniscus, distortion of the orifices anteriorly and the mucocutaneous junction, and hyperemia. Posterior eyelid margin hyperemia, accompanied by telangiectasias, is one of the most common pathological signs, observed mainly in the elderly [3]. Telangiectasias, seen as abnormal, dilated capillaries visible on the posterior eyelid margin, contribute to the vicious cycle of inflammation in the pathogenesis of DED via the secretion of inflammatory cytokines, making eyelid margin vascularity an important player in MGD pathophysiology [4]. Although there are many literature reviews on MGD, to the best of our knowledge, there is no review on the role of eyelid margin vascularity in MGD diagnosis and management. This review aims to provide a simplified and logical approach for the pathophysiology, clinical associations and importance, newer imaging techniques, and emerging treatment methods targeting eyelid margin vascularity.

## Review

Pathophysiology of eyelid margin vascularity in meibomian gland dysfunction

The increased eyelid margin vascularity in MGD is regulated by multiple molecular mechanisms. Inflammatory cytokines, such as interleukin-1 (IL-1) and tumor necrosis factor-alpha (TNF-α), are released in response to meibomian gland obstruction and microbial overgrowth [5,6], leading to the activation of endothelial cell proliferation and contributing to increased vascular permeability and dilation of the normal existing eyelid margin vessels. In addition to direct damage to the vascular endothelium, persistent inflammation stimulates neoangiogenesis, which promotes the formation of abnormal blood vessels in the eyelid margin.

Matrix metalloproteinases (MMPs), particularly MMP-9, play significant role in telangiectasias formation. Extracellular matrix components are degraded in response to the presence of MMPs [7]. This degradation influences the structural integrity of blood vessels, leading to vessel dilation and leakage. MMPs are also involved in the secretion of different pro-inflammatory cytokines, initiating a vicious cycle of inflammatory responses [8].

Localized inflammation of the meibomian glands is triggered by microbial overgrowth at the eyelid margin. Demodex mites are the most common pathogens. Induced inflammation causes elevated cytokine levels, promoting vascular changes. Staphylococcus species, especially *Staphylococcus aureus* and coagulase-negative Staphylococcus, are prevalent in the meibomian glands and eyelid margins. *Staphylococcus aureus* produces crude extracts that induce hyperkeratinization in the meibomian gland ducts and acini, leading to overexpression of keratin 1 (Krt1), which promotes glandular obstruction and dysfunction [9]. The activation of NF-κB and AIM2/ASC inflammasome signaling pathways by *S. aureus* further exacerbates inflammation, contributing to blood vessel dilation (telangiectasias) on the eyelid margin. The ability of bacteria to switch between α-GlcNAc and β-GlcNAc glycosylation in vivo enhances their virulence and resistance to immune clearance, amplifying inflammation and vascular abnormalities [10]. *Staphylococcus aureus* forms a biofilm on the eyelid margin, which protects it from antibiotics and immune defenses. This biofilm not only increases the production of matrix metalloproteinases (MMPs) but also releases lipases and toxins that degrade meibum, leading to saponification and further inflammation, which promotes telangiectasias development [11].

Another critical aspect in telangiectasias formation is oxidative stress caused by inflammatory responses [12]. Reactive oxygen species (ROS) increase endothelial cell apoptosis, damage vascular endothelial cells, and promote vascular dysfunction. This oxidative stress environment, in combination with inflammation, stimulates a cascade of vascular remodeling processes, including increased cell proliferation, extracellular matrix deposition, and angiogenesis [13]. These vascular remodeling changes ultimately contribute to the development of telangiectasias.

The ability of vascular endothelial growth factor (VEGF) to increase vascular permeability and promote inflammatory cell migration to the ocular surface constitutes VEGF, an important player in the chronic inflammation observed in MGD [14]. Emerging treatments targeting VEGF have shown efficacy in reducing inflammation and promoting restoration of meibomian gland function. This action of anti-VEGF treatment suggests that VEGF not only serves as a biomarker for inflammation in MGD but also represents a potential therapeutic target.

Association With Rosacea

Rosacea shows a strong correlation with MGD and ocular surface abnormalities, since approximately 30% to 50% of patients diagnosed with rosacea present with concurrent MGD [15]. Recently, Yang et al. observed a unique vascular pattern on the upper eyelid skin, characterized by a linear or branching arrangement that often formed a typical “spider web” appearance, accompanied by telangiectasia at the eyelid margin, using a high-resolution facial imaging system (VISIA) [16]. A higher prevalence of significant meibomian gland loss, reduced gland area, and decreased gland density has been observed in rosacea patients than in controls [17,18]. Ocular erythema, lid margin alterations, dry eye, impaired glandular expressibility, abnormal secretion patterns, and telangiectasia have been described as common symptoms and signs. Moreover, common inflammatory pathways are implemented in the pathogenesis of both conditions, leading to ocular surface dysfunction. The non-specific clinical manifestations and minimal cutaneous signs may lead to the underdiagnosis of rosacea and MGD.

Aging and Vascular Changes

Various changes in eyelid margin and meibomian gland anatomy have been associated with aging. Increased eyelid margin vascularity, telangiectasia, and hyperkeratinization have been described [19]. The decline in skin collagen and elastin production in combination with thinning of the blood vessel wall and reduced vascular tone make the underlying vessels more susceptible to dilation and formation of telangiectasias [20]. Aging-related diseases, such as diabetes and hypertension, may exhibit low-grade chronic inflammation with an increased release of inflammatory cytokines [21]. During menopause in women, reduced estrogen levels affect vascular health and blood vessel reactivity [22]. Toxic environmental factors, along with reduced regenerative capacity, act cumulatively in the compromise of skin integrity and increased visibility of eyelid margin telangiectasias.

Racial and Demographic Variations

Eyelid margin telangiectasias are more commonly observed in older patients, and the lower eyelid is more frequently affected. An epidemiological study found that patients with meibomian gland dysfunction (MGD) and eyelid margin telangiectasias were significantly older than those without these abnormal vessels [23]. Concerning gender, females tend to exhibit a higher prevalence of eyelid margin telangiectasias and shorter tear film break-up time (TBUT) values, influencing the clinical presentation of MGD [24].

Blumberg et al. noted that darker skin pigmentation was associated with lower visualization of eyelid margin vascularization than lighter pigmentation [25]. It is possible that darker pigmentation blocks the visibility of abnormal vessels, leading to underdiagnosis in individuals with darker skin. Additionally, higher rates of anterior blepharitis and higher grades of eyelid margin telangiectasias have been reported in Caucasians [26].

Clinical assessment of eyelid margin vascularity

Slit lamp examination is the most commonly used method in clinical practice for evaluating the anterior segment and eyelid margin. Through this technique, which is available in all ophthalmology units, a physician can evaluate, apart from telangiectasias, accompanying hyperemia, hyperkeratinization, or meibum expression. All of these clinical signs are necessary for the diagnosis and follow-up of patients with MGD. Modern slit lamp systems offer the capability to acquire digital photographic images of the eyelid margin, which can be stored and shared for diagnosis or follow-up [27].

At many clinical departments, a standardized grading system is often used to quantify the telangiectasias severity [28]. The grading system most commonly used for eyelid margin telangiectasias is a grading scale, where: Grade 0: No visible telangiectasia; the lid margin appears normal, with redness equivalent to that of control skin. Grade 1: Mild telangiectasia; slight redness or a few dilated vessels are present but not prominent. Grade 2: Moderate telangiectasia; more pronounced redness with several visible dilated vessels. Grade 3: Severe telangiectasia; marked redness and numerous engorged vessels are clearly visible, indicating significant vascular changes.

The grading system for eyelid margin telangiectasias shows some differences between the upper and lower eyelids, mainly owing to anatomical and physiological variations. At the upper eyelid tarsus, there are about 30-40 meibomian glands compared to 20-30 at the lower tarsus. In addition, the upper eyelid margin is thicker than the lower eyelid, resulting in lower visibility of the more abundant abnormal vessels at the upper eyelid margin.

In order to improve the visualization of abnormal vessels, red-free (green) filter has been proposed. The red-free filter blocks longer wavelengths of light, particularly in the red spectrum, leading to better contrast between blood vessels and surrounding tissues. Under red-free light, blood vessels appear almost black against a uniformly dark green background, facilitating the identification of subtle vascular changes. Despite the ease of evaluation and clinical practice, there is a high possibility of subjectivity in grading because the evaluation relies on human factors.

Biomarkers and imaging

Since the invention of optical coherence tomography (OCT) in 1991, it has been widely used for in vivo examination of the eye, especially the fundus. OCT is based on the principle of coherence scanning interferometry, during which reflected and scattered light is collected from tissues at different depths, and the collected scanning duration and reconstructed signals are analyzed to construct two- or three-dimensional (3D) images [29]. The evolution of swept-source optical coherence tomography angiography (SS-OCTA) has allowed the visualization and quantification of the eye vasculature, especially of the posterior segment. In MGD, SS-OCTA can provide high-resolution three-dimensional imaging of the eyelid margin vasculature, offering detailed visualization of blood flow and vascular networks and identifying subtle changes in vascularization associated with MGD [30]. Due to the longer wavelength (1050 nm) used in SS-OCTA, deeper tissue penetration and better visualization of vascular structures below pigmented tissues are possible. Moreover, the ability to quantify lid margin blood flow density (LMBFD) makes the evaluation of eyelid margin vascular changes more objective compared to the traditional slit lamp microscopy, presenting high repeatability and reproducibility [31].

The non-invasive nature of this technique reduces patient discomfort and risk of complications, as no dye injection or physical contact with the eye is required. With extremely high scan speeds of up to 400,000 A-scans per second, SS-OCTA technology accelerates the imaging process time, allowing for efficient data acquisition even in patients with limited cooperation [32].

LMBFD seems to correlate significantly with clinical parameters of MGD, such as the Ocular Surface Disease Index (OSDI) [33], fluorescein tear break-up time (FTBUT), and meibomian gland expressibility (ME). This feature makes SS-OCTA a valuable tool for evaluating disease severity, follow-up, and guiding treatment strategies.

Advanced algorithms used by SS-OCTA, such as OCTA ratio analysis (OCTARA), have been developed to reduce motion artifacts and improve the detection sensitivity of abnormal vessels, thereby enhancing the accuracy of vascular imaging and quantification [34].

Role in disease severity

Eyelid margin vascularity is highly correlated with the clinical and biochemical severity of MGD, since elevated lid margin vascularity is linked to more severe MGD symptoms and a higher ocular surface inflammatory response. Kim et al. reported that LMBFD, measured with SS-OCTA, correlates positively with MGD clinical severity, presenting higher OSDI scores and more intense corneal fluorescein staining. In addition, elevated interleukin-4 (IL-4), interferon-gamma-γ (IFN-γ), TNF-α, and matrix metalloproteinase-2 (MMP-2) levels were observed in the presence of increased eyelid margin vascularity, suggesting increased inflammatory activity in MGD [35].

Although there is no direct evidence to confirm the direct transfer of inflammatory cytokines from telangiectasias to the meibomian glands, there is substantial evidence suggesting that inflammation at the eyelid margin, including telangiectasias, contributes to MGD through the release of inflammatory mediators and recruitment of immune cells, which can indirectly affect meibomian gland function [36]. Eyelid vascularity has been shown to correlate with higher levels of inflammatory cytokines, such as IL-17A and matrix metalloproteinase-2 (MMP-2), which are known to contribute to ocular surface inflammation [35]. Furthermore, treatments targeting vascularity, such as intense pulsed light (IPL) therapy and anti-VEGF injections, have been effective in reducing lid margin vascularity and improving MGD symptoms, indicating a significant role of vascularity in the pathophysiology of the disease.

Subclassification of meibomian gland dysfunction (MGD) based on vascularity

Currently, there is no widely recognized classification of MGD based on the presence of eyelid margin telangiectasias. However, the classification of MGD as telangiectatic or non-telangiectatic is based on the observed different diagnostic criteria, prognostic value and treatment response [28]. Recently, the TFOS DEWS III (2025) report, superseding DEWS II (2017), provides updated global consensus guidelines on dry eye and MGD [37,38]. It emphasizes eyelid margin vascularity as a diagnostic hallmark, with telangiectasias and hyperemia serving as clinical signs of MGD severity. DEWS III refines diagnostic algorithms, recommending slit lamp evaluation and non-invasive imaging (e.g., meibography, OCTA) to quantify vascular changes alongside gland morphology. These updates position vascularity as a key parameter for stratifying MGD phenotypes, guiding personalized treatment. For example, pronounced vascularity may indicate a need for anti-inflammatory or vascular-targeted therapies, aligning with heterogeneity studies. Unlike the 2011 TFOS MGD report, which focused on gland-centric definitions, DEWS III integrates vascularity into a broader diagnostic framework, enhancing its clinical utility.

The telangiectatic form is mainly characterized by telangiectasias on the eyelid margin, often accompanied by inflammation and other signs of MGD, such as altered meibum secretion and gland dropout. Main clinical features include redness, swelling, and visible blood vessels at the eyelid margin. Ocular rosacea and other inflammatory conditions affecting the eyelid margin are strongly associated with this form. Management often involves anti-inflammatory therapies, such as intense pulsed light (IPL) to target telangiectasias, topical corticosteroids, or immunomodulating agents, such as cyclosporine. Lid hygiene and warm compresses are also recommended.

In contrast, non-telangiectatic MGD is characterized by the absence of visible telangiectasias. It is typically associated with obstructive or hyposecretory forms of MGD. Patients may present with meibomian gland obstruction, altered meibum quality, or gland dropout without significant eyelid margin vascularity. Treatment focuses on relieving gland obstruction via thermal pulsation, lid hygiene, and meibomian gland expression. Anti-inflammatory treatments are not necessary unless inflammation is present.

Consequently, the presence or absence of eyelid margin telangiectasias can contribute to differentiating between rosacea-associated MGD and other forms of MGD. Telangiectatic MGD is often more challenging to treat due to its association with chronic inflammation and systemic conditions like rosacea. This classification is important for addressing the underlying causes and for customizing the treatment for each patient.

Targeted therapies on eyelid margin vascularity

Anti-inflammatory Medications

The effectiveness of corticosteroids in reducing inflammation by inhibiting the release of pro-inflammatory cytokines and suppressing immune cell activation is important for restoring MG function, leading to improved lipid secretion and tear film stability [39]. Moreover, the suppression of inflammatory mediators, such as VEGF, by corticosteroids reduces vascular dilation and permeability and eyelid margin vascularity [40]. Despite the high effectiveness of corticosteroids, careful monitoring of corticosteroid use is crucial because of the potential risk of increased intraocular pressure (IOP), cataract formation, and skin thinning [41]. Therefore, short-term use of low-potency topical corticosteroids (e.g., loteprednol etabonate or fluorometholone) is generally recommended for managing acute inflammation in telangiectatic MGD [42]. In clinical practice, corticosteroids are often used in combination with other treatments, such as antibiotics (e.g., doxycycline) or omega-3 supplements, to address both inflammation and MGD [43].

Cyclosporine is also an effective treatment option for telangiectatic MGD. Like corticosteroids, cyclosporine promotes the downregulation of inflammatory processes, improves gland function, and reduces eyelid margin telangiectasias [44,45]. Inhibition of T-cell activation and reduction of pro-inflammatory cytokine production (e.g., IL-2, TNF-α, and IFN-γ) breaks the vicious cycle of chronic inflammation in telangiectasia formation. Additionally, cyclosporine promotes lacrimal gland function, leading to increased tear production, which is particularly beneficial for patients with DED associated with MGD [8]. Unlike corticosteroids, cyclosporine is ideal for prolonged use because it is well-tolerated and is not associated with corticosteroid side effects [46]. The most widely used and studied concentration is 0.05% (Restasis®, Allergan Inc., Irvine, CA, USA), while the 0.1% concentration (Cequa®, Sun Pharmaceutical Industries Ltd., Mumbai, India) exhibits higher potency, making it suitable for not responding to 0.05% patients [47]. Recently, the introduction of nano-cyclosporine formulations in clinical practice has offered better clinical outcomes, as they exhibit superior efficacy in improving key dry eye parameters, such as tear film break-up time (TBUT), corneal staining scores, and meibomian gland secretion scores [48].

Recently, Wong et al. reported that topical spironolactone can normalize meibomian gland function and reduce inflammation and eyelid margin vascularity [49]. Spironolactone acts as a competitive antagonist of androgen receptors, a mineralocorticoid receptor inhibitor, and reduces pro-inflammatory cytokines. By addressing hormonal and inflammatory imbalances, spironolactone promotes healthier meibum secretion and improves the tear film quality.

*Anti-Vascular Endothelial Growth Factor* *(VEGF)*

Bevacizumab is an emerging treatment choice for MGD management, targeting abnormal eyelid margin telangiectasias. Jiang et al. reported an effective intra-meibomian gland injection of bevacizumab, reducing eyelid margin vascularity and improving meibomian gland function, which indicates the key role of abnormal vascularity of the eyelid margin in MGD pathophysiology [24]. In the operating room, participants received an intra-MG injection of bevacizumab (150 μL, 2.5 mg/0.1 mL) with a 29-G needle (1 mL; BD, Franklin Lakes, NJ, USA) at multiple sites in the MGs in both upper and lower eyelids in regions of severe telangiectasia. Clinical improvements in eyelid margin vascularity, conjunctival injection, and meibomian gland expressivity were observed as early as one-week post-injection and were sustained for up to three months. Tear film stability was also improved by increasing TBUT and reducing OSDI scores. No serious local or systemic side effects were observed, indicating a favorable safety profile. Recently, Tantipat et al. compared intra-meibomian gland injections to topical bevacizumab eye drops [50]. While the intra-meibomian gland injection of bevacizumab was superior and more effective in reducing telangiectasia grading and improving corneal staining, the less invasive approach of eye drops makes this method suitable for patients who cannot tolerate injections.

Intense Pulsed Light

Intense pulsed light (IPL) is a relatively novel treatment choice for telangiectatic MGD management that focuses on eyelid margin telangiectasia elimination. IPL improves meibomian gland function through photothermolysis, reducing telangiectasias and inflammatory mediators [51]. Photothermolysis includes photocoagulation of abnormal vasculature and thrombosis of telangiectatic vessels via the emission of polychromatic light (500-1200 nm) absorbed by hemoglobin in blood vessels [52]. It enhances gland microstructure, upregulates anti-inflammatory cytokines, and promotes collagen synthesis. Heat from the IPL may improve meibum expression, eradicate Demodex, stabilize the tear lipid layer, reduce tear osmolarity, and restore gland function [53].

Different filters can be used to improve safety and efficacy. Acne filters (notch type) block mid-spectrum wavelengths (600-800 nm), enhancing selective vascular destruction while minimizing epidermal damage [54]. Treatment protocols use fluences of 10-14 J/cm², and usually three to four sessions of two to four weeks intervals are usually needed with direct eyelid application. The clinical outcome of IPL (telangiectasia reduction and symptom relief) is retained for three to six months post-treatment, although repeated sessions may be needed to preserve outcomes [55]. A randomized controlled trial (RCT) demonstrated significant improvements in MGD symptoms and gland function after IPL, with notable reductions in telangiectasia scores and improved meibomian gland secretion scores. These results support IPL mechanism of selective photothermolysis, targeting pathological vessels to reduce vascular-driven inflammation [52].

Regarding safety, no serious adverse events have been reported, except a transient discomfort [46]. Peira et al. confirmed IPL efficacy in reducing MGD symptoms (e.g., OSDI score, TBUT) and vascularity, particularly in rosacea-associated cases, with minimal adverse events [56]. However, some case reports have documented rare but serious ophthalmic side effects of IPL, including macular hole formation, uveitis, synechiae, photophobia, and pupillary abnormalities [57]. Despite the proven efficacy of IPL in telangiectatic MGD, it seems to be less effective in Fitzpatrick skin types V-VI, because more light energy is absorbed in darker pigmented skin. Moreover, IPL seems to be less effective in older patients and those with severe gland atrophy [54]. The main limitation of this method is the need for expensive specialized equipment and repeated sessions, which limits its widespread use.

Overall, IPL therapy is a safe and effective treatment option for reducing telangiectasias in MGD, with robust evidence supporting its role in disrupting pathological angiogenesis and inflammation. Although acne filters and combination protocols may enhance outcomes, individualized treatment plans and maintenance sessions are critical for sustained benefits. Future research should standardize treatment protocols and explore their long-term durability.

Future directions and research gaps

As there are no universally accepted diagnostic criteria, the evaluation of eyelid margin vascularity differs across different clinical settings. Establishing a standardized methodology could improve the repeatability and reliability of clinical evaluation, which is needed to compare research findings, diagnosis, and monitoring of patients with telangiectatic MGD.

Molecular pathways involved in the pathogenesis of meibomian gland dysfunction associated with eyelid margin vasculature have not been fully investigated. Current literature suggests that alterations in eyelid margin vascular changes may affect gland health via inflammatory processes. Cytokine profiles, angiogenesis, and the role of vascular endothelial cells in MGD could be future research topics to reveal potential therapeutic targets. Customized treatment protocols are going to be the future of MGD management, where tailored treatments will be based on individual eyelid margin vascular and meibomian gland profiles. Detailed objective evaluation of eyelid margin vascularity with advanced imaging techniques, such as SS-OCTA, is a useful tool that provides data for customized treatment plans and the clinical effectiveness of the provided treatment. In conclusion, addressing these research gaps is vital for advancing our understanding of the role of eyelid margin vascularity in MGD and the development of better treatment choices.

## Conclusions

Eyelid margin vascularity is a critical biomarker of MGD, indicating chronic inflammation and meibomian gland dysfunction. Molecular mechanisms, such as cytokine release (e.g., IL-1, TNF-α), microbial overgrowth, and oxidative stress, promote vascular remodeling and ocular surface damage. Elevated VEGF and MMP-9 levels further promote neoangiogenesis and inflammation. Advanced imaging tools, such as SS-OCTA, enable the objective and quantitative evaluation of vascular changes, showing a strong correlation with clinical severity. Clinically, the classification of MGD into telangiectatic and non-telangiectatic forms improves prognostic accuracy and customizes the treatment. Telangiectatic MGD, often associated with rosacea or aging, is more benefited from anti-inflammatory treatments, such as IPL, anti-VEGF agents, and immunomodulators. Emerging treatments, including topical spironolactone and nano-drug formulations, are promising for normalizing gland function and reducing vascularity. However, demographic variations, such as skin pigmentation and age-related anatomical changes, complicate diagnosis and require standardized grading systems to avoid underdiagnosis. Future studies should focus on elucidating cytokine networks, endothelial cell interactions, and genetic predispositions in order to identify novel therapeutic targets. Additionally, addressing disparities in diagnostic accuracy across skin types and refining imaging protocols (e.g., SS-OCTA) will improve clinical applicability and reproducibility. The future of MGD management may involve personalized treatment based on vascular and gland profile via advanced imaging and biomarkers. By targeting eyelid margin vascularity when needed, clinicians can disrupt the inflammatory cascade, preserve ocular surface health, and improve patient quality of life.
